# The second generation metagenomic sequencing of cerebrospinal fluid for diagnosis of non-eschar scrub typhus

**DOI:** 10.1093/omcr/omae198

**Published:** 2025-03-20

**Authors:** Shujing Li, Sufang Zhang, Tao Zhou, Zhe Ge, Yi Li, JiYing Zhang, Yifei Zhu, Shaoying Li

**Affiliations:** 920 Hospital Clinical College, Kunming Medical University, 1168 Chunrong West Road, Chenggong District, Kunming City, Yunnan Province 650500, China; Department of Respiratory and Critical Care Medicine, 920th Hospital of Joint Logistics Support Force PLA, Kunming No. 212, Daguan Road, Xishan District, Yunnan Province, 650500, China; Department of Respiratory and Critical Care Medicine, 920th Hospital of Joint Logistics Support Force PLA, Kunming No. 212, Daguan Road, Xishan District, Yunnan Province, 650500, China; Department of Respiratory and Critical Care Medicine, 920th Hospital of Joint Logistics Support Force PLA, Kunming No. 212, Daguan Road, Xishan District, Yunnan Province, 650500, China; Department of Respiratory and Critical Care Medicine, 920th Hospital of Joint Logistics Support Force PLA, Kunming No. 212, Daguan Road, Xishan District, Yunnan Province, 650500, China; Department of Respiratory and Critical Care Medicine, 920th Hospital of Joint Logistics Support Force PLA, Kunming No. 212, Daguan Road, Xishan District, Yunnan Province, 650500, China; 920 Hospital Clinical College, Dali University, 22 Wanhua Road, Xiaguan Town, Dali, Yunnan Province, China; Department of Respiratory and Critical Care Medicine, 920th Hospital of Joint Logistics Support Force PLA, Kunming No. 212, Daguan Road, Xishan District, Yunnan Province, 650500, China

**Keywords:** scrub typhus, sequence analysis, *Orientia tsutsugamushi*, doxycycline

## Abstract

Tsutsugamushi disease, is an infectious disease transmitted by ticks and caused by the rickettsiella. It is characterized by eschar, fever, rash, and flu-like symptoms. However, diagnosing atypical cases without an eschar and with negative Weil-Felix test results poses a significant diagnostic challenge. This study presents a noteworthy case of non-eschar typhus which was effectively diagnosed using advanced next-generation sequencing (mNGS) of cerebrospinal fluid (CSF) samples.

## Introduction

A 77-year-old male patient presented with high fever, headache, and neurological symptoms, which strongly suggested central nervous system involvement. However, no eschar was observed, and the results of the serological Weil-Felix test were negative. Through the utilization of mNGS analysis, Rickettsial DNA was detected in CSF, confirming the diagnosis of tsutsugamushi infection. This case serves to emphasize the potential of mNGS as an invaluable tool for diagnosing atypical infectious diseases. By providing a comprehensive and unbiased assessment of the pathogen landscape, mNGS transcends the limitations of conventional diagnostic techniques. Its ability to accurately and promptly identify pathogens allows for early establishment of a precise diagnosis and initiation of targeted antimicrobial therapy, thus leading to favorable clinical outcomes.

## Case report

**Figure 1:**
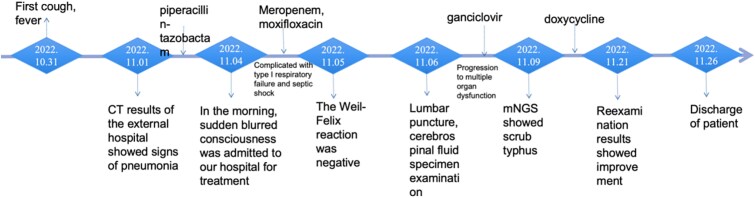
The process of patient admission.

**Figure 2:**
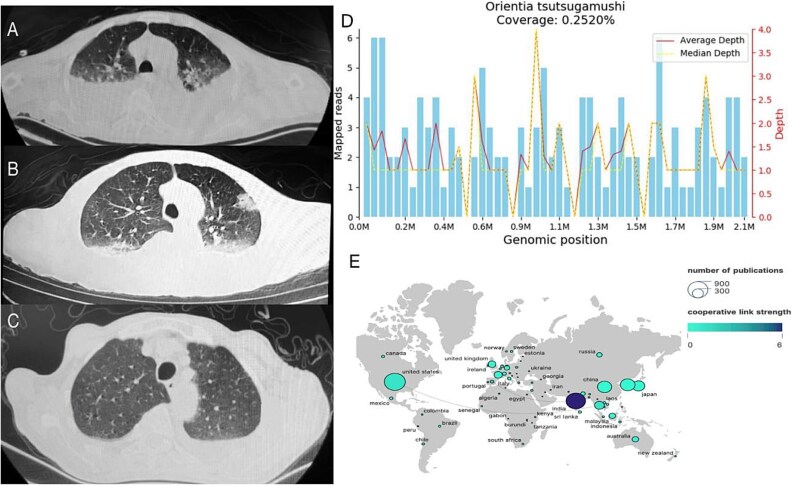
Chest CT examination, mNGS sequencing results and visual map of studies on tsutsugamushi in different countries.(A) on November 7, a high resolution chest CT showed double lung pneumonia, moderate pleural effusion and partial atelectasis. (B) on November 11, chest CT examination revealed double lung pneumonia and decreased pleural effusion. (C) on November 22, chest CT examination again showed that the pneumonia infiltration was significantly absorbed and the pleural effusion was significantly less than before. (D)analysis results of mNGS sequencing. The coverage of referenced *Orientia tsutsugamushi* genome was 0.25%. (E) visual maps of international cooperation between countries studying *O. tsutsugamushi*. 8442 items were found in the web of science using keywords like "scrub typhus", "tsutsugamushi fever", "*O. tsutsugamushi*", "tsutsugamushi disease", "tropical typhus", "epidemic typhus", and "tsutsugamushi pneumonia".

A 77-year-old male patient from Yunnan, China had recently traveled to Guangxi Province one week before the onset of the disease. On October 27th, 2022, the patient began to develop symptoms such as fever, chills and sweating. He was treated with oral ibuprofen suspension and intravenous infusion of piperacillin tazobactam. However, these measures have not led to significant improvements. Due to a sudden high fever, blurred consciousness, lethargy, and slurred speech on November 4th, the patient was admitted to the hospital for treatment. Upon physical examination, the heart rate was 96 beats per minute. The respiratory rate was 25 breaths per minute, blood pressure was 93/70 mmHg, transcutaneous oxygen saturation was 78%, and body temperature fluctuated between 37.5–40°C. There were no rash or subcutaneous hemorrhages on the skin and mucosa throught the body, and no swelling or tenderness in the superficial lymph nodes. Auscultation revealed diminished breath sounds over both lung fields. Positive Babinski's and Gordon's signs were observed in the right lower extremity, along with neck stiffness and positive Kernig's and Brudzinski's signs, which raised suspicion. Furthermore, the levels of alanine aminotransferase were at 250 U/l, aspartate aminotransferase at 467 U/l, direct bilirubin at 7.5 μmo1/l, lactate dehydrogenase at 991 U/l, α-hydroxybutyrate dehydrogenase at 666 U/l, and CRP at 125.40 mg/l. The serum Weil-Felix test revealed a Proteus OXK antibody level of 1:80. A chest CT on November 7th showed thickening of interlobular septa, thickening of bronchial vascular bundles, multifocal ground glass shadow, partial atelectasis, and bilateral pleural effusion (Fig. 2A). Consequently, upon the initial diagnosis of septic shock and type I respiratory failure, empirical anti-infective therapy was administered with moxifloxacin and meropenem. Additionally, antipyretic therapy was administered with ibuprofen. However, due to the unknown cause of the infection, there has been limited improvement in symptoms. At the same time, various tests were conducted to diagnose infectious pulmonary diseases, including *Mycobacterium tuberculosis*, Legionella, Mycoplasma, Chlamydia, Adenovirus, and so on. However, all the test results came back negative. Results of autoimmune antibodies tests in all patients were negative, which did not support the diagnosis of autoimmune diseases. Blood and sputum cultures also showed no bacterial growth. On November 6th, with the consent of the patient and his family, a lumbar puncture was performed to examine the cerebrospinal fluid. The cerebrospinal fluid was light yellow and transparent, with chloride 120 mmol/l, glucose 3.26 mmol/l, IgG 0.470, ink staining negative, positive Pandy's test, lactate dehydrogenase 67 umol/l, adenosine deaminase 2.8 umol/l, and cerebrospinal fluid protein 807.4 mmol/l. The count of nucleated cells in the cerebrospinal fluid was 6 × 10^6^ cells/l, and no bacterial growth was observed even after five days of incubation. Moreover, the quantitative detection of mycobacterium tuberculosis nucleic acid was negative, so viral meningitis was initially suspected, and ganciclovir antiviral therapy was administered. However, chest ultrasound and CT (Fig. 2B) on November 11st revealed an increase in pleural effusion, and the cranial CT scan revealed a symmetric reduction in white matter density surrounding the bilateral lateral ventricles, whereas no significant abnormalities were observed in other brain parenchymal structures. Additionally, the patient exhibited high fever, disturbed consciousness, and a white blood cell count below 4^*^10^9^/l, indicating further progression to systemic inflammatory response syndrome (SIRS). After a multidisciplinary consultation, considering the severe infection of the central nervous system (CNS), metagenomic second generation sequencing (mNGS) was performed on the cerebrospinal fluid, revealing the presence of oriental tsutsugamushi. Detailed pathogen detection results are shown in Fig. 2D. Combined with a history of travel prior to the fever, the patient was prescribed doxycycline 100 mg twice a day on November 9th. After 14 days of treatment, the patient regained consciousness and all vital signs, including body temperature, transcutaneous oxygen saturation, and heart rate, returned to normal. Additionally, there were no negative signs observed during brain stimulation. Laboratory results showed elevated platelets, decreased erythrocyte sedimentation and C-reactive protein (CRP), elevated albumin, decreased liver enzymes, suggesting that the treatment was effective. The chest CT on November 21st showed a decrease in fluid volume compared to the previous one (Fig. 2C), partial lobe reexpansion. The patient was discharged in stable condition on November 26th. Detailed laboratory test results and the process of patient admission are shown in Table 1 and Fig. 1.

**Table 1:** The patient's laboratory test results.

	05.Nov	07.Nov	08.Nov	10.Nov	13.Nov	16.Nov	21.Nov	23.Nov
Na+ (mmol/l)	133↓	135↓	129↓		138		140	
Ka+ (mmol/l)	4.12	4.15	3.56		3.52		3.26↓	
Scr (μmol/l)	56↓	54↓	51↓		42↓		44↓	
TP (g/l)	58.9↓	57.1↓	67.3		69.7		58.8↓	
GPT (U/l)	232↑	192↑	181↑		70↑		34	
CRP(mg/l)	153.40↑		25.30↑		140.70↑		34.50↑	
PCT(ng/ml)	1.19↑		0.14		0.3		0.23	
WBC (^*^109/l)	7.10	6.78	54.4	5.76	5.5	3.33↓	4.54	5.68
NEUT# (^*^109/l)	5.59	4.1	3.8	4.2	4.13	2.06	3.01	4.44
LYMPH# (^*^109/l)	1.15	2.6	1.31	1.08↓	0.82↓	0.93↓	1.17	0.78
RBC (^*^1012/l)	4.38↓	3.91↓	3.23↓	3.34↓	3.22↓	3.58↓	3.10↓	3.35↓
PLT (^*^109/l↓)	61	40↓	62↓	86↓	214	230	214	357
D-dimer (mg/l)	18.32↑		11.29↑	4.74↑	1.16↑	1.91↑		
PO2 (mmHg)	65.0↓	61.0↓	67.0↓	69.0↓				
PCO2 (mmHg)	29.0↓	34.0↓	40.0	43.0				

## Discussion

Scrub typhus, also known as chigger mite fever, is an acute febrile disease transmitted by chigger mites. The main endemic areas for scrub typhus, also known as "Tsutsugamushi Disease Triangle", are located in the "Tsutsugamushi Disease Triangle" of East Asia, Southeast Asia, South Asia, northern Australia, and Pacific island countries, but it has now spread worldwide due to globalization. These regions contribute to almost 50% of global research on this topic (see Fig. 2E). Among them, the necrotic damage caused by mite bites is referred to as an eschar, which is one of the characteristic manifestations used to diagnose scrub typhus. These lesions contain a high bacterial load and can be transmitted through the lymphatic system and bloodstream, causing systemic symptoms such as fever, rash, elevated levels of C-reactive protein, and abnormal liver enzymes [1]. Evidence suggests that scrub typhus poses a risk to more than 1 billion people each year, resulting in more than 1 million deaths [2]. The mortality rate for complex scrub typhus involving the central nervous system can be as high as 13.6%, and severe cases without proper rickettsial treatment can have a mortality rate of up to 30% [3]. Early recognition and diagnosis of tsutsugamushi is essential for subsequent treatment and prevention of disease progression. Diagnosis can be confirmed through clinical history, travel history, the presence of fever, eschar or ulcer, and a Weil-Felix titer of 1:160 or higher. While this test has a wide range of applications, its sensitivity and specificity are poor. The World Health Organization (WHO) recommends indirect immunofluorescence (IFA) as the gold standard diagnostic method [4]. However, this technique lacks uniformity and results in titer variability among laboratories. PCR, on the other hand, is quite accurate in diagnosing tsutsugamushi disease. Obtaining the best sample for diagnosis can be challenging because it requires a biopsy of eschar tissue, which is not always present in every patient. Although the ELISA is a preliminary and widely employed technique for detecting rickettsial infections, the timing of sample collection is crucial for the accuracy of these tests. These are the difficulties in diagnosing non-escharic tsutsugamushi disease. However, mNGS exhibits greater sensitivity than traditional culture methods, especially in detecting uncommon pathogens. Furthermore, mNGS has a broad coverage spectrum, allowing it to detect mixed infections and assist in the precise selection of appropriate antibacterial agents [5].

In this case, the patient has a history of residing in subtropical regions, experienced fever and impaired consciousness, and no eschar was found on the skin or mucous membranes throughout the body. The clinicians were appropriately concerned about the possibility of scrub typhus. But the negative results of the Weil-Felix test hindered a timely diagnosis. The results of the cerebrospinal fluid cell count and bacterial culture did not indicate any bacterial infection. Additionally, the blood cultures were negative, and despite receiving anti-infection and antiviral therapy, the patient did not show significant improvement. Ultimately, the mNGS results supported tsutsugamushi, and the patient was successfully treated with doxycycline.

In summary, when tsutsugamushi disease affects the CNS, it is commonly associated with symptoms such as fever and headache. Additionally, there may be positive meningeal irritation. Furthermore, previous studies have confirmed the diagnosis of scrub typhus through the use of ELISA and PCR testing on cerebrospinal fluid samples [6]. When there is no typical eschar lesion and no positive etiological findings, the condition may be misinterpreted as viral meningitis, leading to a delay in treatment. When conventional diagnostic methods cannot pinpoint specific pathogens, the judicious use of metagenomic next-generation sequencing (mNGS) can elucidate the diagnosis, enable prompt targeted treatment, and optimize patient prognosis.
